# Geoseq: a tool for dissecting deep-sequencing datasets

**DOI:** 10.1186/1471-2105-11-506

**Published:** 2010-10-12

**Authors:** James Gurtowski, Anthony Cancio, Hardik Shah, Chaya Levovitz, Ajish George, Robert Homann, Ravi Sachidanandam

**Affiliations:** 1 Department of Genetics and Genomic Sciences, Mount Sinai School of Medicine, 1425 Madison Avenue, New York, NY 10029, USA; 2 International NRW Graduate School in Bioinformatics and Genome Research, Center for Biotechnology (CeBiTec), Bielefeld University, 33594 Bielefeld, Germany

## Abstract

**Background:**

Datasets generated on deep-sequencing platforms have been deposited in various public repositories such as the Gene Expression Omnibus (GEO), Sequence Read Archive (SRA) hosted by the NCBI, or the DNA Data Bank of Japan (ddbj). Despite being rich data sources, they have not been used much due to the difficulty in locating and analyzing datasets of interest.

**Results:**

Geoseq http://geoseq.mssm.edu provides a new method of analyzing short reads from deep sequencing experiments. Instead of mapping the reads to reference genomes or sequences, Geoseq maps a reference sequence against the sequencing data. It is web-based, and holds pre-computed data from public libraries. The analysis reduces the input sequence to tiles and measures the coverage of each tile in a sequence library through the use of suffix arrays. The user can upload custom target sequences or use gene/miRNA names for the search and get back results as plots and spreadsheet files. Geoseq organizes the public sequencing data using a controlled vocabulary, allowing identification of relevant libraries by organism, tissue and type of experiment.

**Conclusions:**

Analysis of small sets of sequences against deep-sequencing datasets, as well as identification of public datasets of interest, is simplified by Geoseq. We applied Geoseq to, a) identify differential isoform expression in mRNA-seq datasets, b) identify miRNAs (microRNAs) in libraries, and identify mature and star sequences in miRNAS and c) to identify potentially mis-annotated miRNAs. The ease of using Geoseq for these analyses suggests its utility and uniqueness as an analysis tool.

## Background

Deep sequencing platforms such as the Illumina's Solexa Genome Analyzer and ABI's Solid, have simplified the generation of large short-read datasets [1]. Many of these datasets are now deposited in publicly-accessible repositories such as the Sequence Read Archive (SRA) at the NCBI [2].

However, a researcher interested in exploring the public datasets is faced with two problems,

• Identifying the right libraries. The short-read datasets are neither uniformly annotated, nor are they organized to make searches easy.

• Analyzing the libraries for features of interest. The sheer magnitude of the data in these datasets poses computational challenges.

Each experiment can result in tens of millions of reads and requires specialized software to conduct proper analysis. The analysis of these datasets can be unwieldy without proper computer hardware and software solutions.

The usual solution to this problem is to map the reads from the datasets to the genome and use these mappings for further analysis. In such a framework, if a sequence needs to be analyzed against the dataset, then the sequence is also mapped to the genome and the intersections of the two sets of mappings are used to infer biology. A popular tool for such analysis is galaxy [3].

In many cases, a user simply wants to look at the representation of a few sequences (genomic fragments or transcripts) within one or more short-read datasets. This is difficult to do using the existing tools without precomputing mappings for every dataset against every potential query sequence.

We can use a suffix-array based technique to precompute indexes of short-read datasets and rapidly calculate coverage over any query sequence. Such queries can be made fast enough to allow a web-based solution, where a client can rapidly request analyses from many remote servers.

Geoseq is a tool that solves the problems of both navigating public sequencing datasets and quickly performing small-scale analyses on these. The Geoseq user-interface provides a controlled vocabulary that makes it easy to locate short-read libraries. The Geoseq analysis service then allows rapidly mapping sequences against the short-read libraries for analysis of genes, miRNAs and other sequence types. We demonstrate its utility in this paper on data from a variety of types of experiments, such as mRNA-seq and sRNA-seq.

## Implementation

We describe here the software underlying Geoseq, as well as the algorithms. The use of the tool is described in the Results section.

### Geoseq algorithms and software overview

Web-based applications have many advantages over desktop software including server-side data management, seamless updates and cross-platform usability. However, one factor that often prevents developers from using the web as an application platform is speed. Geoseq attempts to find a balance between speed and precision by implementing a tile-based, exact-match search algorithm.

### Geoseq algorithm implementation

We use the pre-existing libfid [4] suffix array library to build indexes of sequencing experiments and later query these datasets to quantify the representation of each tile in the dataset. Suffix arrays require a costly one time indexing of the sequence data, allowing for subsequent rapid exact-match searches. SRA datasets are downloaded and run through a pipeline where suffix arrays are built and description information is saved for later location of the dataset. This pipeline is automated and can be run periodically to update the local database. The process ensures the latest public datasets are available for analysis.

Suffix Arrays allow substring lookups to be conducted very quickly at the expense of flexibility, providing only the ability to lookup exact matches. This is suboptimal for sequence alignment where gaps and deletions are often necessary.

Geoseq uses exact matches of sub-strings from a string to find inexact matches. First, a query of length *n *is split into k-mers (also called tiles) of length k, specified by the user. A k-mer at a given position *i *is the substring of length *k *starting at *i*. The tiles are all possible k-mer tiles that cover the sequence. These tiles are then searched individually using the exact-match suffix array algorithm. By changing the tile-size, the user can control the precision of the matches pulled from the dataset.

Choosing a smaller tile-size will cause smaller substrings of the query to be searched against the dataset. The smaller the substring, the greater the chance it will match the reads by random chance. In essence, by decreasing the tile-size specificity is decreased while sensitivity is increased. If tile-size is increased, sensitivity is decreased but specificity is increased. By modulating the tile-size the user can find the best balance of precision and misalignment that best suits his or her needs.

#### Browser

The SRA from NCBI associates a set of xml files with each submission that contains information about the experiment, runs, instrument, samples, and submitters. However, the website provides little categorical organization of the information, and the user is forced to rely on the 'search' box, which is not easy to use. Our database schema succintly characterizes the datasets, and associates with each the following information,

• Organism: Signifies the species that is the source of the samples used to generate the dataset.

• Experiment Type: Datasets are also specified by the type of sequencing experiment, using the following sub-categories,

1. Genomic DNA: Whole Genome Sequencing as well as targeted sequencing efforts.

2. ChIP-Seq: Sequencing of immuno-precipitated chromatin.

3. RNA-Seq: Transcriptome studies.

4. sRNA-Seq: short RNAs sequencing experiments (e.g. miRNAs, piRNAs)

• Tissue Type: The source of the DNA/RNA when it can be identified. (e.g Heart, Lung, Breast, Stem-cell, various cell lines).

### Hardware and software architecture

**Figure 1:**
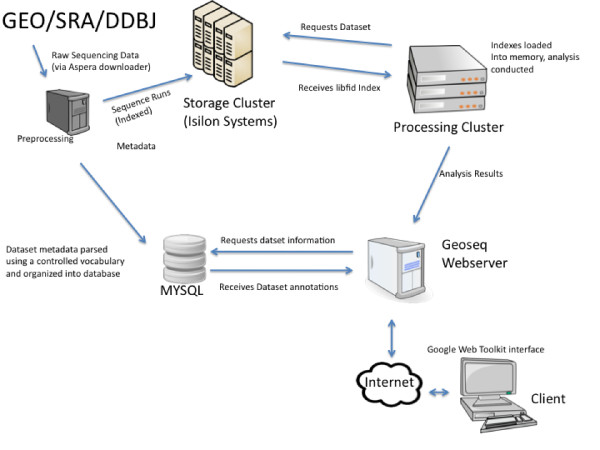
**Geoseq Architecture - Deep sequencing datasets are retrieved from public datastores such as NCBI's SRA**. The metadata gets processed and organized in a database. The sequences are indexed using a suffix array. The suffix array indexes are saved to a storage cluster. The client can browse or search the metadata via a browser. When the client selects a dataset of interest, an analysis request is submitted to the processing cluster through a JSON service. The processing cluster retrieves the suffix array indexes from the storage cluster and performs the analysis. All results are returned to the browser either as graphs or as downloadable links.

An overview of the system is presented in Figure 1. The software infrastructure consists of three components.

• A pre-processing/population component which downloads and pre-processes datasets,

• A browsing component that allows the user to view the datasets that are available, and

• An analysis component which allows datasets to be searched with specific queries and presents the results through the browser.

The pre-processing component is a pipeline that collects sequencing and meta data and organizes them in a database. This is automated by a collection of perl scripts which populate a MySQL database. These scripts are run periodically to ensure our repository is synchronized with SRA's database. The sequencing files are indexed using the libfid (ANSI-C) library. The index files of a sequencing experiment can range in size from 10 gb to 20 gb. Because of their large size, these libraries are stored on an Isilon Storage cluster which is connected over a 10 gbe network to a cluster of processing machines.

The front-end allows the user to browse the datsets available in our repository. This component is written in Java using the Google Web Toolkit http://code.google.com/webtoolkit/. The site is designed to make browsing the datsets as painless as possible.

Once the libraries are selected in the front-end, their indexes are loaded into RAM on the processing server. To spread requests evenly over the processing cluster, a python wrapper was written around the C search functions and deployed as a JSON-webservice onto every processing machine. On the user's submission, the front-end website randomly selects a processing machine to perform the analysis. Once the analysis is complete, the results are returned to the user's browser, formatted for display.

## Results

Here, we will describe Geoseq and apply it to different types of sequencing datasets.

### Tool description

**Figure 2:**
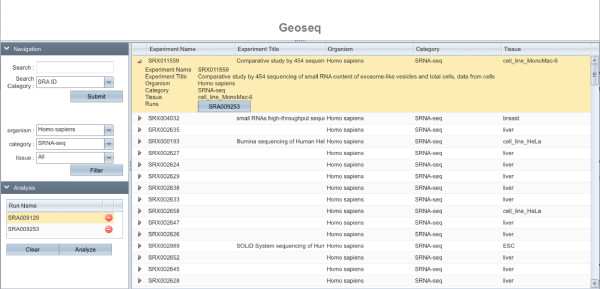
**Dataset Browsing/Selection**. The upper-left pane allows filtering of the datasets. The main pane shows the results of the filtering. Details of the run can be expanded by clicking on the arrow before the name. If the read is indexed, a button appears that select the dataset for analysis and places the name in the lower left pane. After the selection is done, cliking on the *Analyze *button will launch the form to build a search query (figure 3). Only pre-indexed libraries can be selected for further analysis.

**Figure 3:**
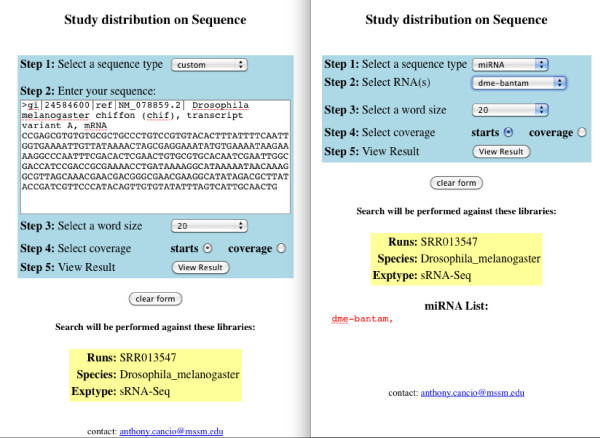
**Query Building**. A box at the bottom of the page confirms the datasets previously selected. The user can either select enter a custom sequence (left) or use a miRNA from our database (right). his will be used to search the previously selected dataset. The result of the analysis is shown in Figures 6 and 7.

Geoseq consists of two parts, a browser (Figure 2) that helps the user identify short-read libraries of interest, and an analyzer (Figure 3) that can be used to query the selected libraries with a user-specified reference sequence.

**Figure 4:**
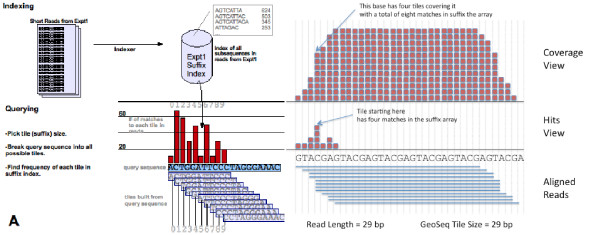
**Geoseq Analysis**. Geoseq uses a tiered process to analyze sequencing data. A. Reads from a deep sequencing experiment are converted into a suffix index for rapid querying. Querying of an input sequence against the experiment is done by breaking the sequence into all possible tiles of a given size and finding the frequency of each tile in the suffix index. B. Visualization of the results can be done in either the Starts View which shows the hits for a tile at the position of the start of the tile, or the Coverage View which integrates hits from all tiles covering a particular position.

**Figure 5:**
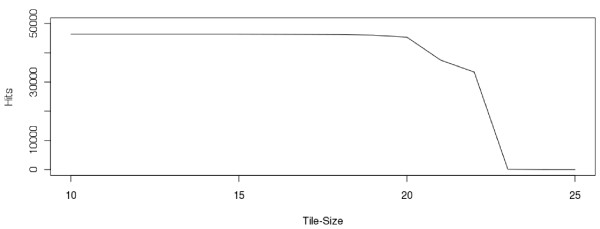
**Choice of Tile-size**. The number of hits for a particular miRNA changes with the tile-size used to query a sRNA-Seq dataset. The size of the tile is varied, while the start of the tile is held fixed at the beginning of the mature sequence of mmu-mir-24-1. At lower tile-sizes, the number of hits corresponds to fragments of the mature sequence that were sequenced. As the tile-size approaches the size of the miRNA, there is a drop-off in hits as there exist fewer reads that span the entire mature sequence. Finally, when the tile-size exceeds the length of the mature miRNA (22-bp), the number of hits drop to zero. The tile-size controls sensitivity and specificity, larger tile sizes increase specificity while smaller tile sizes increase sensitivity.

Geoseq's analysis software tiles the input sequence in steps of one, using a user-specified tile-size and searches for matches of each tile against a library of short-reads (Figure 4). The choice of tile-size determines the output, for example, while searching for miRNAs in a small-RNAseq experiment, choosing a tile-size greater than the mature miRNA length will result in no matching tiles (Figure 5). Thus, optimum tile-size is dependent on the information that is desired and the experiment under consideration.

#### Browser for short read datasets

The browsing interface (Figure 2) allows the user to identify datasets of interest, by filtering libraries on the basis of organism, type of experiment and type of tissue. The vocabulary for the filtering criteria was created by studying the meta-data provided by the researchers. Manual curation was used to classify libraries that have not been properly annotated.

#### Analysis of libraries with a user-supplied sequence

The primary goal of Geoseq is to provide a means of determining how well a sequence of interest is represented in a dataset. Users can choose to query against known genes and miRNAs or against a custom sequence that they upload. These search sequences are broken up into tiles (of a user-specified size) and used to query a dataset. The number of times each tile occurs in the dataset of interest is reported back in the form of a Lightweight Genome Viewer graph [5] and corresponding downloadable spreadsheet.

#### Additional features for sRNA-seq data

In the case of miRNAs, the user can generate a summary report for the library that provides the number of hits in the dataset for all known miRNAs in the species. This is useful in characterizing the miRNA spectra represented in the library. A complementary feature allows the user to pick a specific miRNA and get a listing of all sRNA-seq libraries that show it expressed.

### Application of Geoseq

We demonstrate the utility of Geoseq by applying it to the exploration of a few short-read sequence datasets.

#### mRNA-Seq

**Figure 6:**
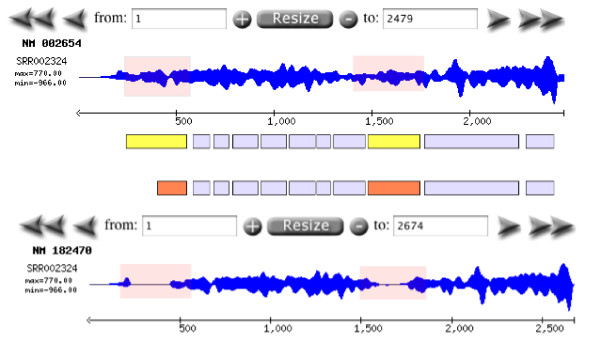
**mRNA-seq**. Using the sequences for splice-variant forms of a gene, NM_182470 and NM_002654 for PKM2, in Geoseq allows identification of the correct version that is expressed in the sample being sequenced. The second isoform, NM_182470, shows gaps in coverage (highlighted) indicating that only the first isoform, NM_002654, is expressed.

mRNA-seq is the deep sequencing of mRNA transcripts from cells (usually isolated using oligo-dT beads). This is a primary method for the analysis of known and novel genes and their isoforms [1,6]. Geoseq allows the users to select one of the known Refseq transcripts or to specify their own sequence and then to visualize the coverage of short reads across it. This allows for measurement of expression across a given transcript and for identification of the particular isoforms that are expressed (Figure 6).

#### ChIP-Seq

ChIP-seq is the deep sequencing of DNA bound to proteins that are immunoprecipitated. This is used to enumerate DNA-protein interaction sites and to identify specific sequence binding-motifs for DNA-binding proteins. Geoseq has several indexed ChIP-seq datasets. The binding of the proteins to DNA can be studied, by analyzing DNA sequence fragments that might contain potential binding motifs against the indexed libraries on Geoseq.

#### sRNA-Seq (smallRNA-Seq)

sRNA-Seq is the deep sequencing of small RNAs (usually shorter than 36 nucleotides) isolated from total-RNA, for the purpose of studying the expression patterns of miRNAs, piRNAs (piwi-binding small RNAs), and other small RNA molecules [7].

Geoseq allows several kinds of analyses for small RNAs.

**Figure 7:**
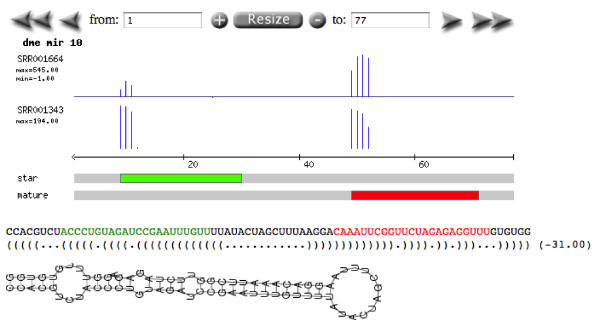
**microRNA Analysis I**. The output of a Geoseq query run on sRNA-Seq libraries. In the *Starts *view, the histogram displays the number of times the k-mer starting at a position was found in the library (there is one histogram per user-selected library). In the *Coverage *view, the numbers represent the sum of contributions for each tile that covers the position. This figure shows the results of miRNA, dme-mir-10 queried against two D. melanogaster libraries, SRR001664 and SRR001343. The tracks below the histograms indicate the positions of known mature and star sequences for mir-10 according to miRBase [8]. Below this is shown the folded structure of the pre-miRNA from RNAfold [15]. In experiment SRR001664, the expression of the mature sequence is greater than the expression of the star, which is the canonical case. However, in library SRR001343, the expression of the canonical star-sequence is similar to the expression of the mature sequence. This suggests that the roles of star and mature sequences may, on occasions, be context-dependent.

1. Identify libraries that contain a particular miRNA. On picking the experiment type sRNA-seq, Geoseq allows the option of filtering libraries by the occurrence of a particular miRNA (Figure 2).

2. Identify expression levels of miRNAs in a particular library. After the selection of the library, in the analysis window, Geoseq will allow the user to generate summary reports of the miRNA expression in the selected library.

3. Identify the mature and star sequences of a miRNA. Using the profiles generated by Geoseq, it is possible to identify the mature and start sequences, as shown in Figure 7.

microRNAs are small non-coding molecules involved in post-transcriptional regulation, among other possible functions. On transcription, the pre-miRNA forms a hairpin, which is processed to a short double-stranded RNA (approximately 22 nucleotides long). One of the strands, called the mature, is biologically active, while the complementary strand, called the star, is degraded. Geoseq holds information (downloaded from miRBase [8]) on microRNAs, such as their pre-miRNA and known mature and star sequences.

#### Identification of mis-annotated microRNAs

**Figure 8:**
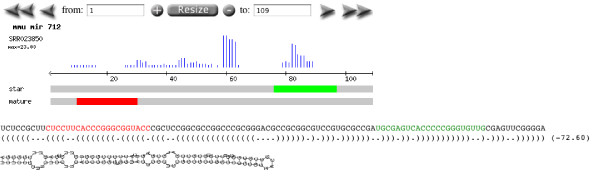
**microRNA analysis II**. Geoseq allows us to identify expression patterns of microRNAs in libraries. Here we show expression for mmu-miR-712 in SRR023850 which does not exhibit the canonical pattern seen for most other microRNA libraries (Figure 7). This was used to identify mis-annotated miRNAs from miRBase that are listed in Table 1.

We demonstrate the application of Geoseq to identify potentially mis-annotated miRNAs. Geoseq can be used to systematically scan the expression patterns of the pre-miRNAs across libraries to detect anomalies. An anomalous, or non-canonical, expression pattern is shown in Figure 8 which is different from what is normally seen for pre-miRNAs as shown in Figure 7. Non-canonical profiles can potentially signify a mistaken identification of another non-coding RNA, such as a snoRNA (small-nucleolar RNA) or tRNA, as a miRNA.

We use the following metric to identify several mis-annotated miRNAs that we list in table 1. Using a tile-size of 20 we assign a value to each position, *i*, equal to the number of occurrences (*N_i_*) of the tile starting at that position in the library. If the total of the number in the mature (*N_m_*, the sum of all *N_i _*in the mature sequence) and number in the star (*N_s_*, the sum of all *N_i _*in the star sequence) sequences is less than 95% of the total over the whole primary miRNA sequence,

**Table 1:** Anomalous microRNAs.

	miRBase id	Species	Libraries	Nature of Anomaly	remarks
1	hsa-miR-619	Human	SRR018353, SRR019624	misannotaton	m. on opp. strand
2	hsa-miR-1975	Human	SRR013571, SRR029124	misannotation	RNY5 ?
3	mmu-mir-546	Mouse	SRR034120, SRR014235	non-canonical	snoRNA ?
4	mmu-mir-1957	Mouse	SRR023850, GSM307159	misannotation	wrong mature
5	dme-mir-929	D.mel	SRR014278, SRR031696	misannotation	mature is star

A list of microRNAs that have either another potential function or incorrectly identified mature and star sequences.

These were analyzed by studying the distribution of reads over the pre-miRNA. (see also Figure 8).

(1) $$\left(N_{m}+N_{s}\right)<0.95*\sum_{i}^{}N_{i}$$

then the miRNA is deemed to be potentially mis-annotated. In order to conclusively prove a mis-annotation, we examine the underlying genomic annotations, study the fold structure, and use sno-RNA predictions to infer the actual function. An example of this is shown in Figure 8 for the expression pattern for mmu-miR-712 in library SRR023850. After examining the annotation of that region in the Genome Browser at UCSC we conclude that this is most likely a tRNA.

This metric has allowed us to identify several microRNAs (a partial list is shown in table 1) whose identification as miRNAs probably need to be revisited.

## Discussion

Geoseq aggregates and organizes libraries of short-read sequencing data allowing users to quickly find datasets of interest and query them using Geoseq's tiled-search algorithm.

### Benefits of the browsing function

A comparison of the browsing function with the interface provided by SRA quickly reveals the advantages of Geoseq. Searching for the term *small RNA mouse[orgn] *in the SRA interface [2], returns 22 libraries. Using Geoseq's interface, a user can filter for organism and Experiment Type (M. musculus and SRNA-Seq) and find over 140 libraries (at the time of writing). These results can be filtered further by tissue type, with the tissue drop-down box populated by existing tissues types for M. musculus and SRNA-Seq in our database. New datasets are continuously being added as they become public. Geoseq organizes the meta-data more efficiently, letting the user access and analyze more of the public data. Geoseq currently contains small RNA libraries for M. musculus(150), H. sapiens(161), D. melanogaster(229) and C. elegans (91). Additionally, there is information on over 23,672 libraries, comprising mRNA-seq and ChIP-seq experiments from a variety of species. The accuracy of the classification and the ability to access these datasets is the result of a manual curation of the libraries. Occasionally, the manual curation has resulted in the correct reclassification of some libraries that are imprecisely described in the original meta-data.

### Comparison of Geoseq analysis to existing tools

The current generation of short-read analysis tools work by building indexes of reference genomes or other target sequences and mapping the short reads to these reference sets. Some of these tools include the new Burrows-Wheeler or suffix-array based tools like SOAP [9], Bowtie [10], and MAQ [11] and older sequence alignment tools such as BLAT [12] and BLAST [13]. In all of these tools, the unit of analysis is a single read and each short read is either mapped back to the reference sequence or is discarded. Coverage for a target sequence of interest is measured by counting the number of reads that align across it.

The deepBase database http://deepbase.sysu.edu.cn/ is a browser-based tool that lets users browse through deep sequencing datasets [14]. DeepBase presents some precomputed analyses (such as read-length distributions, nucleotide biases etc.) and links to relevant papers and snoRNA predictions (for cases where the miRNAs have been misclassified). In comparison, Geoseq has a more comprehensive collection of libraries and allows more criteria for filtering, and the analyses offered is more versatile, by allowing the users to explore the datasets using their own sequences.

Geoseq turns the paradigm of mapping reads to a reference on its head. We build suffix-array indices for each short-read sequencing dataset (instead of the reference sequence datasets) and then use a tiling approach to find coverage across a target sequence of interest. Our algorithm takes a user specified target sequence and breaks it into smaller substrings (tiles) that are independently searched against a short read index. Coverage for a target sequence is measured by the number of times each tile is found in the short read index.

Specificity and sensitivity are two important variables in estimating target sequence coverage. Specificity here corresponds to the number of allowed mismatches in a read-alignment. Current short read analysis tools such as Blast allow the user to increase the specificity of a search by reducing the number of allowed alignment mismatches. In Geoseq, more specific matches can be ensured by increasing the tile-size. Sensitivity, in the context of short-read analysis, refers to the ability to pick up more partial matches of short reads to target sequence. In Geoseq, reducing the tile-size allows shorter, partial matches and increases the sensitivity (Figure 5). The current short read analysis tools offer no method to modulate sensitivity, as the unit of analysis must always be an individual short read. Geoseq's tiled-search approach thus allows a way to simultaneously tune sensitivity and specificity of short-read mapping.

Geoseq's tiled-search algorithm also has strong benefits in a web-based environment where speed is of the utmost importance. Geoseq indexes short read databases using suffix array algorithms that trade an expensive pre-processing operation for the benefit of fast subsequent searches.

### Limitations of Geoseq

Geoseq is best used for the analysis of small sets of sequences, against a short-read dataset. If large sets of sequences need to be analyzed, or the genomic distribution of the short-read dataset is desired, then it is better to perform the traditional analyses of mapping the short-reads to the genome and using that as the basis of analysis.

The suffix-array indices for short-read datasets consists of many large files (10-50 GB). Creating and storing public indices en-masse is thus prohibitively resource intensive. We have addressed this by indexing a cross-section of the available data and allowing users to request that particular data be indexed. In addition, the analysis of datasets is memory intensive as each suffix-array must be loaded into RAM before it can be queried. To keep the load on the processing cluster manageable, we currently limit the number of datasets the user may query at one time. Though there are multiple servers handling analysis requests, the web-based service is still limited by network latency, and the presence of multiple concurrent users may impact the time it takes for results to be returned. The time taken for a single query can range from 30-120 seconds, depending on the size of the sequencing library and the input sequence.

## Conclusions

Geoseq has made public datasets available for exploration and analysis. Useful datasets can be located using a controlled vocabulary and links are given for accessing the source of the datasets. A subset of these datasets have been downloaded and pre-processed. The pre-processed datasets can be queried with user-supplied sequences. We have demonstrated Geoseq's utility by using it to analyze microRNAs against several sRNA-seq libraries, as well as isoform expression in mRNA-seq.

We plan to continue improving the classification of libraries, based on user-feedback. In the future, more sophisticated searches, such as those based on MeSH terms or on gene expression profiles will be allowed, by preprocessing the sets for mRNA expression profiles and adding additional annotations. We do not currently index every library in the public domain due to constraints of space and resources, but we plan to address this incrementally by allowing users to request indexing of particularly interesting data.

## Availability and requirements

Geoseq is browser-based and publicly accessible through the internet at http://geoseq.mssm.edu. There are no restrictions on academic use. Geoseq has been tested on several browsers and works on Safari, Firefox and Internet Explorer.

## Authors' contributions

RS came up with the general idea, the details were fleshed out by JG. JG programmed the analysis software, the user-interface and several back-end components, AC developed the initial version, as well as the software used to return the results. RS designed the database and several back-end components. RH. independently developed libfid and helped us use it in geoseq. HS helped develop the software and infrastructure. AG helped with comparisons to other tools and applications to public datasets. JG and RS wrote the manuscript. All authors read and approved the final manuscript.
